# Correlates of Physical Activity of Students in Secondary School Physical Education: A Systematic Review of Literature

**DOI:** 10.1155/2019/4563484

**Published:** 2019-02-19

**Authors:** Yulan Zhou, Lijuan Wang

**Affiliations:** School of Physical Education and Sport Training, Shanghai University of Sport, Shanghai 200438, China

## Abstract

**Background and Objective:**

Several reviews have summarised studies on secondary school students' moderate-to-vigorous physical activity (MVPA) in physical education (PE), but no systematic review with semiquantitative assessment has been conducted to specifically identify the correlates of their MVPA. This review aims to systematically summarise the existing literature, which investigated correlates of MVPA of secondary school students during their PE lessons.

**Methods:**

A systematic search using ERIC, SPORTDiscus, PubMed, PsycINFO, Academic Search Premier, and Web of Science was conducted to identify the correlates of the MVPA of secondary school students in PE. Studies were eligible if they were English published articles and examined the association with MVPA during secondary school PE lessons and cross-sectional and prospective longitudinal quantitative studies. Two reviewers independently examined the articles, assessed their methodological quality, and performed data extraction. The correlates of MVPA were synthesised and further assessed semiquantitatively.

**Results:**

Fifty-five studies were identified to correlate with secondary school students' MVPA in PE lessons. Further analysis only included 43 studies (78.2%) that were of medium and high quality by methodological quality assessment. Out of 54 variables identified from these medium and high-quality studies, 11 were consistently associated with the MVPA. Sex (boys), ethnicity (White), class gender (boys-only), PE activities (team games), lesson location (outdoors), expectancy beliefs, subjective task values, and enjoyment were consistently and positively associated with MVPA. Other variables, namely, class gender (girls-only), PE activities (movement activities), and lesson context (knowledge), were consistently and negatively related to MVPA.

**Conclusions:**

Interventions focusing on the consistent variables are needed to build active lesson time in PE. This review also provides insights for future research.

## 1. Introduction

Physical activity (PA) offers physiological and psychological benefit [1]. Adolescence is a critical time to develop PA patterns which extend to adulthood [2]. However, a large proportion of adolescents worldwide is physically inactive [3]. School physical education (PE) is recognized as a key opportunity for improving PA amongst adolescents for two reasons [4]. PE provides opportunities for children and adolescents to accumulate moderate-to-vigorous PA (MVPA) [5]. PE also aims to provide children and adolescents fundamental movement skills, knowledge, and active attitude for lifetime PA [6].

The United States Department of Health and Human Services [4] and the United Kingdom Association for Physical Education [7] have recommended that school students should engage in MVPA for at least 50% of PE lesson time. However, two reviews by Fairclough and Stratton [8] and Hollis et al. [9], which summarised studies concerning the MVPA level of secondary school students (approximately 10–18 years) in PE classes, found that students spend between 27% and 47% of PE time in MVPA. The findings showed that the MVPA of secondary school students in PE lessons failed to meet the 50% of PE lesson time recommendation.

Understanding the factors influencing MVPA during PE lessons is particularly important for the design of more effective interventions. Many studies have examined the correlates of MVPA in secondary school PE [10, 11]. However, only one review by Fairclough and Stratton [8] analysed 40 relevant studies until 2005 and identified some factors, such as intervention design, activity type, sex, skill level, and motivation which influence the MVPA of secondary school students in PE classes. Numerous works studying the correlates of secondary school students' MVPA during PE classes have been conducted since 2005, and these studies were not summarised and reviewed. Therefore, this review aims to systematically summarise studies on the correlates of secondary school students' MVPA during PE classes until 2018 and identify variables contributing to their MVPA. The findings will aid in developing effective interventions to improve the PA of students and provide insights for PE teaching practice and future studies.

## 2. Methods

### 2.1. Search Strategy

**Figure 1:**
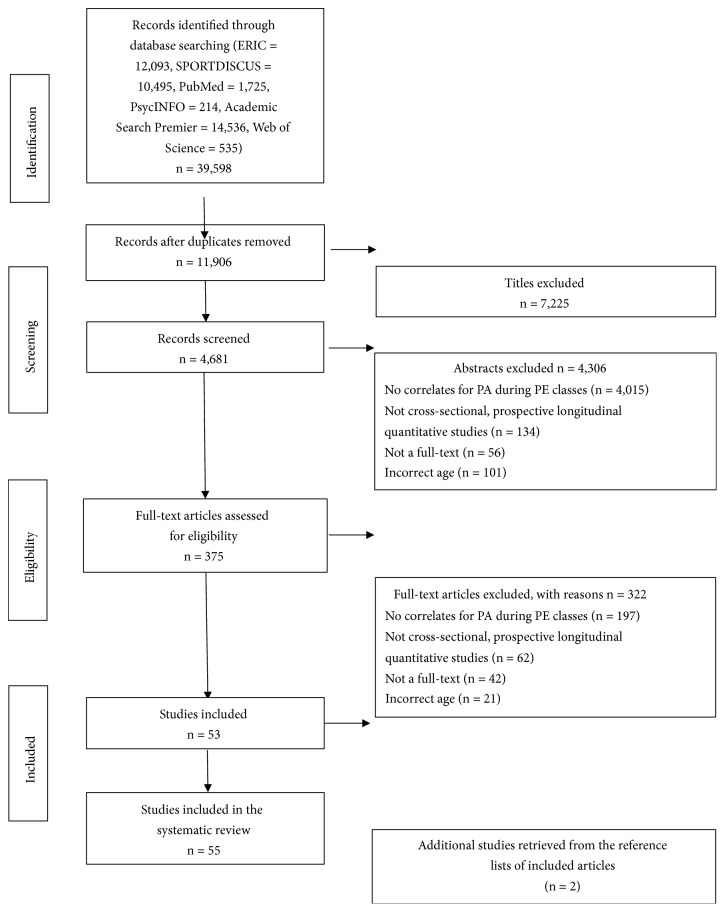
Flow diagram of literature research results.

The process and reporting of this review adhered to the Preferred Reporting Items for Systematic Reviews and Meta-Analysis (PRISMA) statement [12]. A literature search of all electronically archived literature published until May 2018 was conducted in six electronic databases, namely, ERIC, SPORTDiscus, PubMed, PsycINFO, Academic Search Premier, and Web of Science. Search terms were based on the combination of three main areas: (1) setting (physical education OR PE OR lesson*∗* OR class*∗*); (2) target population (youth*∗* OR adolescent*∗* OR teenager OR child*∗* OR student*∗*); (3) measurement terms (physical activit*∗* OR PA OR MVPA OR exercise*∗* OR health behavior OR motor activi*∗*). The search strategies used for each database were shown in the Supplementary Materials (Table S1). In addition, reference lists of all included studies were manually searched for additional relevant papers. After the removal of duplicates and analyses of the titles and abstracts, 375 records remained (Figure 1).

### 2.2. Inclusion and Exclusion Criteria

The inclusion and exclusion criteria were as follows. (1) Articles examining variables for their association with PA during school PE classes were included; those focusing on other topics were excluded. (2) Cross-sectional and prospective longitudinal quantitative studies which examined associations between PA and other variables were covered. Qualitative studies, case reports, and expert opinion were disregarded. Intervention studies were also excluded from this review because they were aimed at determinants instead of correlates of MVPA. According to Bauman et al. [13], determinants reflected a cause and effect relationship, and correlates described a correlation between two variables. (3) Only published peer reviewed journal, with full-text articles in English until May 2018 were included, thereby excluding unpublished articles, conference proceedings, and dissertations. (4) Articles focusing only on general secondary school students (middle [i.e., approximately 10–14 years of age; Grades 6–8]) or high school [i.e., approximately 12–18 years of age; Grades 7–12]) were covered. Following the systematic literature search, the identified articles were determined by two reviewers (Yulan Zhou, Lijuan Wang) to independently check whether the screening procedure was consistent with the inclusion and exclusion criteria. A third reviewer (Yuanyuan Hu) was consulted in case of disagreement. The final number of articles identified in the review was 55. Figure 1 summarises the process of the literature research.

### 2.3. Methodological Quality Assessment

The criteria for assessing the qualities of studies were adapted from the Strengthening The Reporting of Observational studies in Epidemiology (STROBE) statement [14] and the McMaster Critical Review Forms for quantitative research [15], which have been used to assess the methodological quality of previous systematic reviews in similar areas [16, 17]. The criteria contained 8 items which covered aspects of study design, sample, study attrition, data collection, and data analysis (see Table S2 in the Supplementary Materials). Two reviewers (Yulan Zhou, Yuanyuan Hu) independently assessed all studies. Each item was scored as 1 (the assessed item was explicitly described and present) or 0 (the assessed item was inadequately described or absent). Any uncertainty and disagreement were resolved by the third reviewer (Lijuan Wang). The score for each study was summed, and the median score for all included study scores was calculated. Article was determined as high quality when it scored above the median score, medium quality when it scored equal to the median score, and low quality when it scored below the median score [18].

### 2.4. Categorisation of Variables

The demographic data of these studies were summarised and included geographical location, participant characteristics, PA measurement methods, and analytical approaches. The geographical location was summarised into the countries where the study was conducted. The participant samples were split into four groups (<100, 100-249, 250-500, and >500). Furthermore, the analytical approaches such as univariate analysis (e.g. t-tests, ANOVAs, and correlations) and multivariate analysis (e.g., linear regression, logistic regression, and structural equation molding) were also presented (Table 1). Studies that used both analytical approaches were numbered in both categories.

Consistent with previous reviews on correlates of children and adolescent PA [19, 20], the potential correlates of MVPA during secondary school PE classes were classified into four categories, namely, demographic and biological variables, instruction-related variables, school physical environment variables and psychological variables on the basis of the subjects, and research content of these studies.

### 2.5. Coding of Analyses

The review contained three types of variables, namely, ordinal (e.g. self-efficacy), continuous (e.g., lesson length), and categorical (e.g., PE activities and lesson contexts) variables. A variety of analysis methods, including correlation, linear regression, logistic regression, and structural equation modeling, were used to determine the association between the ordinal, continuous variables, and MVPA. The column (Table 2) ‘related to MVPA' indicates a significant relationship between these variables and students' MVPA. The codes ‘+' and ‘−' indicate positive and negative direction of association, respectively. The column ‘unrelated to MVPA' indicates a nonsignificant association between these variables and students' MVPA. With regard to categorical variables (e.g., PE activities), t-test, ANOVA, and multivariate ANOVA (MANOVA) were used to analyse the difference in MVPA amongst multiple variables (e.g., team games, individual games, movement activities and individual activities). For studies focusing on these variables, multiple associations were recorded and summarised under a general heading (e.g. PE activities). The column ‘related to MVPA' indicates significant differences in students' existing MVPA. The codes ‘+' and ‘−' for the categorical variables represent the most and least MVPA time amongst these variables. If not, it was coded as ‘unrelated to MVPA'.

Following a previous review by Sallis et al. [20], variables that appeared in less than three comparisons were not described and discussed in the text. However, these variables were included in the tables to better understand the procedure of variable screening in this review. Semiquantitative assessment [20] was adopted to determine the consistency of different types of potential correlates in this review. This semiquantitative procedure provided additional objective evidence beyond reporting of narrative results only [16]. Variables reported in less than three studies were coded as ‘No Description (ND)'. For variables appearing more than three times, the directions of associations were based on the rules drawn up by Sallis and colleagues [20]: 0%–33% of the association in a similar direction was considered to have no association and coded as “0”; 34%–59% of the association in a similar direction was defined as indeterminate or inconsistent and coded as ‘?'; 60%–100% of the association in a similar direction was regarded as consistent and coded as either ‘+ +' (positive) or ‘− −' (negative). This cut-off coding was used in previous reviews [16, 17, 19].

## 3. Results

### 3.1. Methodological Quality

Agreement in the methodological quality assessment was 87.3% between the two reviewers (Yulan Zhou, Yuanyuan Hu) and achieved full consensus with the help of a third reviewer (Lijuan Wang). The Supplementary Materials (Table S3) provide the results of the methodological quality assessment per study. For the study design, only five (9.1%) studies used a longitudinal/prospective study design. For the sample component, nearly one-third of studies (17 studies, 30.9%) used a representative sample, and the majority of studies (42 studies, 76.4%) describe the participant characteristics in detail. For the study attrition, 13 studies (23.6%) clearly described the response rate and met the criteria. For the data collection, most of the studies used valid measures of PA (50 studies, 90.9%) and reliable measures of the related factors (44 studies, 80.0%). For the analyses component, most of the studies (49 studies, 89.1%) used appropriate statistical analysis and accounted for potential confounders in analysis, and 16 studies (29.1%) reported a power calculation. The median score of all 55 included study scores was 4 (range: 1-7), and 22 studies (40.0%) were rated above the median score and were categorised as high quality; 21 studies (38.2%) were scored as being equal to the median score and described as medium quality, and the remaining 12 studies (21.8%) scored below the median and were classified as low quality. In order to remove the influence of studies with greatest risk of bias, the 12 studies with low quality were excluded from this review [21, 22]. Thus, the final 43 studies were summarised and discussed in the present review.

### 3.2. Description of Studies

Table 1 summarises the distribution of geographic location, sample size, PA measurements, and analytical approaches. Forty-three studies were included in this systematic review, majority of which were conducted in the USA (23 studies, 53.5%). The majority of studies (27 studies, 62.8%) had <250 samples. Objective measures including accelerometers, HR monitors, pedometers, and direct observation measures were adopted in most of the studies (41 studies, 95.3%). Thirty-seven studies (86.0%) used univariate analyses to evaluate the association between variables and students' MVPA during PE lessons, and 20 studies (46.5%) reported multivariate analyses results.

### 3.3. Correlates of Students' PA in Secondary PE Classes

Table 2 summarises studies on the correlates of MVPA of medium- and high-quality students (i.e., 43 studies). A total of 54 variables were identified from these studies. Thirty variables (55.6%) had been investigated three or more times. Very few of these variables (11 variables, 36.7%) were consistently correlated with students' MVPA in all comparisons, and 14 variables (46.7%) were found to have no association. Only five variables (16.6%) were classified into the “inconsistent” category.

#### 3.3.1. Demographic and Biological Variables

A total of 23 studies found seven demographic and biological variables that correlated to students' MVPA time during secondary school PE classes. Five variables, namely, sex, school level, grade, BMI, and ethnicity factors, were studied three or more times. Sex was the most frequently studied correlate (16 studies), and boys were consistently found to be more active than girls (in 75% of the comparisons). The most consistently supported finding in this group was that White students were more active than Black students in PE classes, and all comparisons supported this conclusion. Grade was significantly related to MVPA time in 50% of the comparisons. Therefore, the relationship was inconsistent. No association was found between two variables (i.e., school level and BMI) and the MVPA of students because only 33% and 20% of the associations were in a similar direction, respectively.

#### 3.3.2. Instruction-Related Variables

Twenty-one studies provided information about 24 instruction-related variables that correlated to MVPA during secondary school PE classes, of which 15 variables, such as class size, class gender (boys-only, coeducational, or girls-only), PE activities (team games, individual games, movement activities, and individual activities), lesson contexts (fitness activities, free play, game play, skill drills, management, and knowledge), and teacher gender appeared in three or more studies. Three types of classes were compared to examine the effect of class gender on students' MVPA. All comparisons supported that students engaged in the most MVPA time in boys-only classes and the least MVPA time in girls-only classes. Four types of PE activities were also grouped together; out of 12 comparisons, team games were reported to accumulate the most MVPA time in 67% of the comparisons. Movement activities (e.g., dance and gymnastics) were reported to accrue the least MVPA time in 83% of the comparisons, thereby confirming the consistency of the association. Amongst different lesson contexts variables, only one variable, knowledge consistently (i.e., 71% of the comparisons) provided the least MVPA time for students. Class gender (coeducational) and lesson contexts (fitness activities and game play) were categorised as inconsistent variables. The other variables, such as class size, PE activities (individual games and individual activities), lesson contexts (free play, skill drill, and management), and teacher gender showed no association with students' MVPA.

#### 3.3.3. School Physical Environment Variables

Nine studies investigated the associations between six school physical environment variables and students' MVPA. However, only one variable (i.e., lesson location) was reported by three or more studies, which found that students consistently (i.e., in 67% of the comparisons) spent significantly more time on MVPA during outdoor lessons than during indoor ones.

#### 3.3.4. Psychological Variables

Sixteen studies identified 17 psychological variables as correlates of students' MVPA time during secondary school PE classes, and 9 of those variables were studied three or more times. These variables included intrinsic motivation, identified regulation, external regulation, amotivation, competence, expectancy beliefs, subjective task values, self-efficacy, and enjoyment. Variables found to have consistent and positive association with MVPA time were expectancy beliefs, subjective task values, and enjoyment, which were supported by 67%, 75%, and 67% of the studies, respectively. Although self-efficacy was significantly associated with MVPA, it was reported in less than 60% of the comparisons, thus making the associations inconsistent. Intrinsic motivation, identified regulation, external regulation, amotivation, and competence were classified as not being associated with students' MVPA because less than 34% of the comparisons were in the same direction.

## 4. Discussion

For this review, we identified 55 studies correlating to secondary school students' MVPA in PE lessons. The results of methodological quality assessment showed that most of the studies reviewed were identified as medium and high quality, and only a small group of studies were categorised as low quality.

Further analysis identified 30 variables that were reported three or more times in the medium and high-quality studies. The results showed few variables (11 variables, 36.7%), namely, sex (boys), ethnicity (White), class gender (boys-only, girls-only), PE activities (team games, movement activities), lesson context (knowledge), lesson location (outdoors), expectancy beliefs, subjective task values, and enjoyment were consistently associated with MVPA, which were in accordance with those found in previous reviews on correlates of PA [17, 20]. The other 14 variables were not associated with students MVPA, which indicated that these correlates may be not important to MVPA. The remaining five variables were placed in “inconsistent” category. It is hard to draw a conclusion from these studies because nearly half the studies found an association and half did not. The few consistent relationships may be attributed to research design, measures, and sample size. According to the descriptive statistics and quality assessment of studies in this review, objective measures (e.g., accelerometer and direct observation) and cross-sectional design were utilized by most of the studies. However, the application of objective measures of MVPA and cross-sectional design may weaken the strength of the relationships between the correlates and the MVPA time of students in PE [63, 64]. Therefore, few consistent relationships were reported in the studies that were included in this review. Another reason for the few consistent associations may be the small sample size. The samples in majority of the studies were limited (27 studies, 62.8%<250 participants). Schönbrodt and Perugini [65] reported that obtaining stable correlations is difficult when sample size is less than 250 participants. A small sample size may inflate the actual significance of results because detecting a specific effect is challenging [66]. This issue contributes to the few consistent associations in this review. Furthermore, most of the studies concerning PA of secondary school students in PE were from Western countries. The few consistent relationships found by this review may be representative of Western countries only rather than worldwide.

Demographic and biological variables which were consistently associated with students' MVPA during secondary school PE lessons were sex and ethnicity. The studies consistently reported that boys were more active than girls. This finding was in line with the previous review by Fairclough and Stratton [8]. Sex was the most studied variable in MVPA level differences in the adolescents' PA literature. Substantial evidence indicated that girls were less active than boys [67, 68]. The main reason was that activeness, bravery, aggressiveness, and perseverance are valued more in boys than in girls while gentleness, kindness, approachability, sensitivity, quietness, weakness, and malleability are valued in girls [69]. Moreover, secondary school students are entering their teenage years, a period characterised by increasing sex differences. During this period, girls may perceive or experience additional barriers to PA due to their physical changes (e.g., widening of the hips, increased fat mass) and thus feel less efficacious in PA [70]. These reasons may also explain the considerable MVPA time of boys in PE classes in the current review. This finding implied that targeted intervention should be considered to increase the MVPA time of girls in PE classes. Regarding the ethnicity variable, White students were consistently more active than Black students but for unclear reasons. Further studies are required to explore the reasons behind this difference.

Class gender, PE activities, and lesson contexts were the three instruction-related variables which were consistently associated with MVPA of secondary school students in PE. Amongst three types of classes, namely, boys-only, girls-only, and coeducational PE classes, the consistent findings were that boys-only classes accrued the most MVPA time and girls-only classes yielded the least. This result was predictable because boys are more physically active than girls in PE classes. With regard to the variable of PE activities, students were consistently found to engage in the most MVPA time in team games and accumulate the least MVPA time in movement activities, which was in accordance with previous review on this topic [8]. Students are more active in team games activities probably because of several continuous body movements and the comparatively large translocation across space when compared with other types of activities [71]. In addition, students easily engage in PA that they perceive as meaningful, enjoyable, and display their abilities [72]. Team games were reported to be fun activities that encouraged students' competence, which may contribute to the most MVPA time of students in team game activities [73]. By contrast, movement activities, such as dance and gymnastics, stimulated the least MVPA time primarily because movement activities address aesthetic appreciation and body control [8]. Finally, the consistent finding for the variable of lesson contexts was that students were the least active during knowledge. Knowledge is possibly an inactive lesson context in which students are likely to merely stand or sit, which may result in the small amount of MVPA time [50]. These findings implied that the government should guide the active lesson plans and implementation to meet the MVPA recommendation. Professional development programs focusing on teaching design and teaching strategies for the promotion of student PA in PE classes should be provided to PE teachers. Moreover, PE teachers should thoroughly and considerately design the class content to be taught, allocate the amount of time spent in different lesson contexts, and implement effective teaching strategies to achieve PE objectives and engage students in additional MVPA time.

The only one school physical environmental variable consistently associated with MVPA was lesson location. Students yielded higher MVPA time during outdoor lessons than indoor lessons. Two reasons may explain the difference. Firstly, outdoor lessons provide available facilities, equipment (e.g., soccer fields and playground) and large play spaces, which may be conducive to gross motor experiences and opportunities for students to be active [74]. Secondly, the natural environments during outdoor lessons positively affect certain aspects of mood, such as increased energy, relaxation, and delight, along with decreases in exhaustion, tension, and depression, which probably increased the MVPA time during PE lessons [75].

In terms of the psychological variables, the results consistently supported the positive role of three psychological variables in MVPA time during secondary school PE lessons, including expectancy beliefs, subjective task values, and enjoyment. Expectancy beliefs and subjective task values are two constructs of expectancy-value theory, which suggested that the expectancy-value model is a useful framework for predicting secondary school students' MVPA engagement in PE classes. Expectancy beliefs refer to beliefs regarding the ability to achieve goals and expectancies for success [59]. Subjective task value is defined as a function of four distinct components, namely, importance, interest, usefulness, and cost [76]. Students become increasingly physically active in PE classes when they consider themselves competent in the activity and viewed the PE classes as important, interesting, and useful. However, some researchers suggested that the influence of these two variables on students MVPA is different. Expectancy beliefs motivate students to engage in a particular task at a given moment, whereas subjective task values influence students' long-term motivation. Furthermore, they have different sensitively levels for content during PE [29]. These findings implied that PE teachers should employ some motivational strategies to enhance students' perceived competence in PE, such as modifying activities to students' ability, helping them to achieve success in class, and providing positive instructional feedback during PE classes. Furthermore, it is also important for PE teachers to present PE activities in novel and meaningful way. Enjoyment is the other psychological factor consistently associated with students' MVPA, which suggested that students are involved in PE activities when they experience happiness and enjoyment in PE. Accordingly, PE teachers should adopt certain strategies (e.g., modifying activities to students' ability and self-directed tasks) to present PE activities more interesting and emphasis students' effort in PE.

## 5. Strengths and Limitations

An extensive and systematically searches have been conducted in multiple databases to identify the literature. Furthermore, this systematic review is the first to use a semiquantitative evaluation to identify correlates of secondary school students' MVPA in PE classes. This evaluation allowed for more information concerning the strength of the evidence of an association.

The present review has several limitations. The first potential limitation of this review is the completeness of the literature search. Although an extensive literature search was conducted to identify all published studies, a few published studies were possibly missed in this review due to the use of keywords other than those used in the current work and unclear titles or abstracts used in these missed studies. The other limitation is the exclusion of non-English published studies. Certain studies that could have added relevant information to the field may have been discarded. Thirdly, lack of longitudinal study design has limited the quality of the studies included in this review. Fourthly, semiquantity evaluation was adopted to analyse the research findings of the studies included. This approach addressed the significance and direction of each association to determine the consistency of reported association and could not assess the strength or magnitude of these associations.

## 6. Conclusion and Recommendations

This review classified the variables that correlate with MVPA as “consistent association”, “inconsistent association”, and “no association” categories. Eleven variables are categorised as “consistent association” with students' MVPA time in PE classes. Amongst them, certain correlates, such as sex (boys), ethnicity (White), class gender (boys-only), PE activities (team games), lesson location (outdoors), expectancy beliefs, subjective task values, and enjoyment, are positively associated with students' MVPA; the other variables, including class gender (girls-only), PE activities (movement activities), and lesson context (knowledge), are negatively related to students' MVPA. These variables provided evidence for possible strategies to intervene aiming at increasing students' MVPA. Variables with “inconsistent” relationships with students' MVPA may be subjected to further detailed study. Variables that have “no association” with students' MVPA should be deemphasised in future studies.

Most of the studies were conducted in Western countries and thus represented the correlates of students' MVPA under Western culture only. Further studies are needed from other countries to identify any cultural difference. In addition, out of the four research domains, that on the influence of school physical environment on students' MVPA has been investigated the least (i.e., 9 studies), further studies concentrating on these variables are needed.

Furthermore, the relationship between a variety of demographic and biological, instruction-related, school physical environment, and psychological variables and students' MVPA during PE lessons have been studied but in isolation. Additional studies may be necessary to explore these variables simultaneously to assist in identifying potential interactions amongst different domains.

Finally, a few demographic factors, such as sex, grade, and BMI, may influence the relationship between the correlates in this review and students' MVPA in PE classes. For example, the association between psychological factors and students' MVPA differed between boys and girls. Therefore, future research should be conducted to study the difference in these associations by the sex, grade, and BMI.

## Figures and Tables

**Table 1:** Descriptive statistics for studies used in the systematic review.

Description	N (%)
*Geographic location*	
United States of America (USA)	23 (53.5)
United Kingdom (UK)	4 (9.3)
Spain	4 (9.3)
Finland	2 (4.7)
Hong Kong	2 (4.7)
Other country	8 (18.6)
*Sample size*	
<100	6 (14.0)
100-249	21 (48.8)
250-500	9 (20.9)
>500	7 (16.3)
*Physical activity measurement*	
Questionnaires	2 (4.7)
Observed/SOFIT	8 (18.6)
HR monitor	10 (23.3)
Pedometers	10 (23.3)
Accelerometer	13 (30.2)
*Analytical approaches*	
Univariate analysis	37 (86.0)
Multivariate analysis	20 (46.5)
*Methodological quality*	
= median score 4	21 (48.8)
>median score 4	22 (51.2)
*Variable categories*	
Demographic/biological variables	23 (53.5)
Instruction-related variables	21 (48.8)
School physical environment variables	9 (20.9)
Psychological variables	16 (37.2)

**Table 2:** Summary of studies of correlates of MVPA during secondary school PE lessons.

Correlates variables	Related to MVPA		Unrelated to MVPA References No	Summary code	
	References No	Asso (−/+)		Asso	% Studies
*Demographic and biological variables*					
Sex (boys)	[10, 23–33]	+	[34–36]	+ +	12/16 75%
	[37]	−			
School level (high school)	[35]	+	[27]	0	1/3 33%
	[36]	−			
Grade	[23, 36, 38, 39]	−	[27, 34]	?	4/8 50%
	[31, 33]	+			
BMI (overweight)	[40]	−	[10, 33, 41, 42]	0	1/5 20%
Skill level (high)	[26]	+		ND	
Cardiorespiratory fitness(healthy)	[43]	+		ND	
Ethnicity (White)	[24, 42, 44]	+		+ +	3/3 100%
*Instruction-related variables*					
Class size	[10, 45]	+	[31, 46–48]	0	2/8 25%
	[49, 50]	−			
Class gender					
Boys-only	[32, 44, 47, 51, 52]	+		+ +	5/5 100%
Co-educational	[48, 52]	+	[44, 51]	?	2/5 40%
	[32]	−			
Girls-only	[32, 44, 47, 48, 51, 52]	−		− −	6/6 100%
Lesson length	[51]	−	[47]	ND	
PE activities					
Team games	[10, 26, 30, 35–38, 40]	+	[27, 31, 47]	+ +	8/12 67%
	[34]	−			
Individual games	[38, 47]	+	[10, 26, 30, 31, 34–37]	0	2/10 20%
Movement activities	[10, 26, 30, 35–38, 40, 47, 53]	−	[31, 34]	− −	10/12 83%
Individual activities	[34, 38, 53]	+	[10, 26, 31, 35, 37, 47]	0	3/10 30%
	[36]	−			
Lesson context					
Fitness activities	[32, 48, 50]	+	[45, 47, 51, 52]	?	3/7 43%
Free play			[32, 45, 47, 48, 50–52]	0	0/7 0%
Game play	[32, 51, 52]	+	[45, 47, 48, 50]	?	3/7 43%
Skill drills	[32, 52]	+	[45, 47, 48, 50, 51]	0	2/7 29%
Management	[32]	−	[45, 47, 48, 50–52]	0	1/7 14%
Knowledge	[32, 47, 48, 50, 51]	−	[45, 52]	− −	5/7 71%
Teacher gender (male)			[47, 48, 50]	0	0/3 0%
Teacher experience	[45]	−		ND	
Teacher type (PE specialist)			[50]	ND	
Teacher behavior					
Fitness promotion			[47]	ND	
Fitness demonstration			[47]	ND	
General instruction	[47]	−		ND	
Observation	[47]	+		ND	
Class management			[47]	ND	
Other tasks			[47]	ND	
*School physical environment variables*					
Lesson location (outdoors)	[45, 48, 50, 54]	+	[10, 47]	+ +	4/6 67%
Accessibility of facilities/equipment	[49]	+		ND	
Large Space	[47]	+	[31]	ND	
Natural environment (Fall)	[54]	+	[47]	ND	
School structure quality			[11]	ND	
School structure quantity			[11]	ND	
*Psychological variables*					
Self-determined motivation	[55]	+		ND	
Autonomous motivation	[46]	+		ND	
Controlled motivation			[46]	ND	
Intrinsic motivation	[56]	+	[2, 57]	0	1/3 33%
Identified regulation			[2, 56, 57]	0	0/3 0%
Introjected regulation			[57]	ND	
External regulation	[2]	−	[56, 57]	0	1/3 33%
Amotivation	[56]	−	[2, 46, 57]	0	1/4 25%
Competence	[39]	+	[25, 57, 58]	0	1/4 25%
Autonomy	[58]	+	[57]	ND	
Relatedness	[58]	+	[57]	ND	
Expectancy beliefs	[29, 59]	+	[28]	+ +	2/3 67%
Subjective task values	[28, 59, 60]	+	[29]	+ +	3/4 75%
Achievement goals	[61]	+		ND	
Self-efficacy	[59–61]	+	[10, 23, 62]	?	3/6 50%
Outcome expectancy	[59]	+		ND	
Enjoyment	[2, 62]	+		+ +	2/3 67%
	[25]	−			

Note: Asso = association.

Variables were coded as ‘no description (ND)' when reported less than three studies; when variables appear more than three times, 60%–100% of the association in a similar direction was coded as ‘+ +' (positive) or ‘− −' (negative); 34%–59% of the association in a similar direction was coded as ‘?' (indeterminate/inconsistent); 0%–33% of the association in a similar direction was coded as ‘0' (no association).

## Supplementary Materials

Supplementary Materials Table S1: a record of the search strategy used for each database. Table S2: methodological quality assessment items. Table S3: methodological quality assessment per quality item and per study.

## References

[1] US Department of Health and Human Services (2018). *2018 Physical Activity Guidelines for Americans*.

[2] Bryan C. L., Solomon M. A. (2012). Student motivation in physical education and engagement in physical activity. *Journal of Sport Behavior*.

[3] Hallal P. C., Andersen L. B., Bull F. C. (2012). Global physical activity levels: surveillance progress, pitfalls, and prospects. *The Lancet*.

[4] U.S. Department of Health and Human Services (2010). *Centers for Disease Control and, National Center for Chronic Disease Prevention and Health Promotion Prevention, and Division of Adolescent and School Health, “Strategies to improve the quality of physical education*.

[5] Lonsdale C., Rosenkranz R. R., Peralta L. R., Bennie A., Fahey P., Lubans D. R. (2013). A systematic review and meta-analysis of interventions designed to increase moderate-to-vigorous physical activity in school physical education lessons. *Preventive Medicine*.

[6] Hills A. P., Dengel D. R., Lubans D. R. (2015). Supporting public health priorities: recommendations for physical education and physical activity promotion in schools. *Progress in Cardiovascular Diseases*.

[7] Association for Physical Education (2015). Health position paper. *Physical. Education, Matters*.

[8] Fairclough S., Stratton G. (2005). Physical activity levels in middle and high school physical education: A review. *Pediatric exercise science*.

[9] Hollis J. L., Sutherland R., Williams A. J. (2017). A systematic review and meta-analysis of moderate-to-vigorous physical activity levels in secondary school physical education lessons. *International Journal of Behavioral Nutrition & Physical Activity*.

[10] Molina-García J., Queralt A., Estevan I., Sallis J. F. (2016). Ecological correlates of Spanish adolescents’ physical activity during physical education classes. *European Physical Education Review*.

[11] Dias A. F., Lemes V. B., Brand C. (2017). Association between school structure and physical activity in physical education class and school recess. *The Revista Brasileira de Cineantropometria e Desempenho Humano*.

[12] Moher D., Liberati A., Tetzlaff J., Altman D. G., The PRISMA Group (2009). Preferred reporting items for systematic reviews and meta-analyses: the PRISMA statement. *Annals of Internal Medicine*.

[13] Bauman A. E., Sallis J. F., Dzewaltowski D. A., Owen N. (2002). Toward a better understanding of the influences on physical activity: The role of determinants, correlates, causal variables, mediators, moderators, and confounders. *American Journal of Preventive Medicine*.

[14] von Elm E., Altman D. G., Egger M. (2008). Strengthening the reporting of observational studies in epidemiology (strobe) statement: Guidelines for reporting observational studies. *Bmj British Medical Journal*.

[15] Law M., Stewart D., Pollock N. (1998). *Guidelines for Critical Review Form – Quantitative Studies*.

[16] Li R., Sit C. H. P., Yu J. J. (2016). Correlates of physical activity in children and adolescents with physical disabilities: A systematic review. *Preventive Medicine*.

[17] Lu C., Stolk R. P., Sauer P. J. J. (2017). Factors of physical activity among chinese children and adolescents: a systematic review. *International Journal of Behavioral Nutrition & Physical Activity*.

[18] Zeng N., Ayyub M., Sun H., Wen X., Xiang P., Gao Z. (2017). Effects of physical activity on motor skills and cognitive development in early childhood: A systematic review. *BioMed Research International*.

[19] Craggs C., Corder K., Van Sluijs E. M. F., Griffin S. J. (2011). Determinants of change in physical activity in children and adolescents: a systematic review. *American Journal of Preventive Medicine*.

[20] Sallis J. F., Prochaska J. J., Taylor W. C. (2000). A review of correlates of physical activity of children and adolescents. *Medicine & Science in Sports & Exercise*.

[21] Dodd K. J., Taylor N. F., Damiano D. L. (2002). A systematic review of the effectiveness of strength-training programs for people with cerebral palsy. *Archives of Physical Medicine and Rehabilitation*.

[22] Lubans D. R., Boreham C. A., Kelly P., Foster C. E. (2011). The Relationship between active travel to school and health-related fitness in children and adolescents: A systematic review. *International Journal of Behavioral Nutrition & Physical Activity*.

[23] Allison K. R., Dwyer J. J. M., Makin S. (1999). Self-efficacy and participation in vigorous physical activity by high school students. *Health Education & Behavior*.

[24] Davis K. L., Wojcik J. R., DeWaele C. S. (2016). A comparison of the fitness, obesity, and physical activity levels of high school physical education students across race and gender. *The Physical Educator*.

[25] Fairclough S. (2003). Physical activity, perceived competence and enjoyment during high school physical education. *Physical Education & Sport Pedagogy*.

[26] Fairclough S., Stratton G. (2005). 'Physical education makes you fit and healthy'. Physical education's contribution to young people's physical activity levels. *Health Education Research*.

[27] Ferreira F. S., Mota J., Duarte J. A. (2014). Patterns of physical activity in portuguese adolescents. *Evaluation During Physical Education Classes through Accelerometry*.

[28] Gråstén A., Watt A., Hagger M. (2015). Secondary school students physical activity participation across physical education classes: the Expectancy-value theory approach. *Physical Educator*.

[29] Gu X. L., Solmon M. A., Zhang T. A. O. (2012). Using expectancy-value model to examine students' physical activity engagement and cardiovascular fitness in physical education. *International Journal of Sport Psychology*.

[30] Lyyra N., Heikinaro-Johansson P., Lyyra M. (2017). Exploring in-class physical activity levels during physical education lessons in Finland. *Journal of Physical Education and Sport*.

[31] Singerland M., Oomen J., Borghouts L. (2011). Physical activity levels during Dutch primary and secondary school physical education. *European Journal of Sport Science*.

[32] Smith N. J., Lounsbery M. A. F., McKenzie T. L. (2014). Physical activity in high school physical education: Impact of lesson context and class gender composition. *Journal of Physical Activity & Health*.

[33] Viciana J., Mayorga-Vega D., Martínez-Baena A. (2016). Moderate-to-vigorous physical activity levels in physical education, school recess and after-school time. influence of gender, age, and weight status. *Journal of Physical Activity & Health*.

[34] Laurson K. R., Brown D. D., Cullen R. W., Dennis K. K. (2008). Heart rates of high school physical education students during team sports, individual sports, and fitness activities. *Research Quarterly for Exercise and Sport*.

[35] Kulinna P. H., Martin J., Lai Q., Kliber A., Reed B. (2003). Student physical activity patterns: Grade, gender, and activity influences. * Journal of Teaching in Physical Education*.

[36] Stratton G. (1997). Children's heart rates during British physical education lessons. * Journal of Teaching in Physical Education*.

[37] Sarradel J., Generelo E., Zaragoza J. (2011). Gender differences in heart rate responses to different types of physical activity in physical education classes. *Motricidad European Journal of Human Movement*.

[38] Gao Z., Hannon J. C., Carson R. L. (2009). Middle school students heart rates during different curricular activities in physical education. *ICHPER-SD Journal of Research*.

[39] Parish L. E., Treasure D. C. (2003). Physical activity and situational motivation in physical education: Influence of the motivational climate and perceived ability. *Research Quarterly for Exercise and Sport*.

[40] Gao Z., Oh H., Sheng H. (2011). Middle school students' body mass index and physical activity levels in physical education. *Research Quarterly for Exercise and Sport*.

[41] Fairclough S., Stratton G. (2006). Physical activity, fitness, and affective responses of normal-weight and overweight adolescents during physical education. *Pediatric exercise science*.

[42] Hannon J. C. (2008). Physical activity levels of overweight and nonoverweight high school students during physical education classes. *Journal of School Health*.

[43] Calahorro-Cañada F., Torres-Luque G., López-Fernández I., Carnero E. A. (2017). Is physical education an effective way to increase physical activity in children with lower cardiorespiratory fitness?. *Scandinavian Journal of Medicine & Science in Sports*.

[44] Hannon J. C., Ratliffe T. (2005). Physical activity levels in coeducational and single-gender high school physical education settings. * Journal of Teaching in Physical Education*.

[45] Gill M., Chan-Golston A. M., Rice L. N., Cole B. L., Koniak-Griffin D., Prelip M. L. (2016). Consistency of moderate to vigorous physical activity in middle school physical education. *Family & community health*.

[46] Aelterman N., Vansteenkiste M., Van Keer H., Van den Berghe L., De Meyer J., Haerens L. (2012). Students’ objectively measured physical activity levels and engagement as a function of between-class and between-student differences in motivation toward physical education. *Journal of Sport & Exercise Psychology*.

[47] Chow B. C., McKenzie T. L., Louie L. (2009). Physical activity and environmental influences during secondary school physical education. * Journal of Teaching in Physical Education*.

[48] Mckenzie T. L., Catellier D. J., Conway T. (2006). Girls’ activity levels and lesson contexts in middle school PE: TAAG baseline. *Medicine & Science in Sports & Exercise*.

[49] Bevans K. B., Fitzpatrick L.-A., Sanchez B. M., Riley A. W., Forrest C. (2010). Physical education resources, class management, and student physical activity levels: A structure-process-outcome approach to evaluating physical education effectiveness. *Journal of School Health*.

[50] McKenzie T. L., Marshall S. J., Sallis J. F., Conway T. L. (2000). Student activity levels, lesson context, and teacher behavior during middle school physical education. *Research Quarterly for Exercise and Sport*.

[51] Dudley D. A., Okely A. D., Cotton W. G. (2012). Physical activity levels and movement skill instruction in secondary school physical education, journal of science medicine in sport. *Journal of Science & Medicine in Sport*.

[52] McKenzie T. L., Prochaska J. J., Sallis J. F., LaMaster K. J. (2004). Coeducational and single-sex physical education in middle schools: Impact on physical activity. *Research Quarterly for Exercise and Sport*.

[53] Fröberg A., Raustorp A., Pagels P., Larsson C., Boldemann C. (2017). Levels of physical activity during physical education lessons in Sweden. *Acta Paediatrica*.

[54] Brusseau T. A., Burns R. D., Fu Y. (2016). Contextual factors related to physical activity during daily middle school physical education. *Journal of Science and Medicine in Sport*.

[55] Lonsdale C., Sabiston C. M., Raedeke T. D., Ha A. S. C., Sum R. K. W. (2009). Self-determined motivation and students' physical activity during structured physical education lessons and free choice periods. *Preventive Medicine*.

[56] Gao Z., Hannon J. C., Newton M., Huang C. (2011). Effects of curricular activity on students’ situational motivation and physical activity levels. *Research Quarterly for Exercise and Sport*.

[57] Pan C.-Y., Tsai C.-L., Chu C.-H., Hsieh K.-W. (2011). Physical activity and self-determined motivation of adolescents with and without autism spectrum disorders in inclusive physical education. *Research in Autism Spectrum Disorders*.

[58] Cox A. E., Smith A. L., Williams L. (2008). Change in physical education motivation and physical activity behavior during middle school. *Journal of Adolescent Health*.

[59] Gao Z., Lee A. M., Kosma M., Solmon M. A. (2010). Understanding students' motivation in middle school physical education: Examining the mediating role of self-efficacy on physical activity. *International Journal of Sport Psychology*.

[60] Gao Z., Newton M., Carson R. L. (2008). Students’ motivation, physical activity levels, & health-related physical fitness in middle school physical education. *Middle Grades Research Journal*.

[61] Gao Z., Lochbaum M., Podlog L. (2011). Self-efficacy as a mediator of children's achievement motivation and in-class physical activity. *Perceptual and Motor Skills*.

[62] Ning W., Gao Z., Lodewyk K. (2012). Associations between socio-motivational factors, physical education activity levels and physical activity behavior among youth. *ICHPER -- SD Journal of Research in Health, Physical Education*.

[63] Bürgi F., Meyer U., Granacher U. (2011). Relationship of physical activity with motor skills, aerobic fitness and body fat in preschool children: A cross-sectional and longitudinal study (Ballabeina). *International Journal of Obesity*.

[64] Wang L. (2017). Using the self-determination theory to understand Chinese adolescent leisure-time physical activity. *European Journal of Sport Science*.

[65] Schönbrodt F. D., Perugini M. (2013). At what sample size do correlations stabilize?. *Journal of Research in Personality*.

[66] Filho D. B. F., Paranhos R., da Rocha E. C. (2013). When is statistical significance not significant?. *Brazilian Political Science Review*.

[67] Fernandes H. M. (2018). Physical activity levels in portuguese adolescents: A 10-year trend analysis (2006-2016). *Journal of Science Medicine in Sport*.

[68] Steenholt C. B., Pisinger V. S. C., Danquah I. H., Tolstrup J. S. (2018). School and class-level variations and patterns of physical activity: A multilevel analysis of Danish high school students. *BMC Public Health*.

[69] Lijuan W., Jiancui S., Suzhe Z. (2017). Parental influence on the physical activity of Chinese children: Do gender differences occur?. *European Physical Education Review*.

[70] Butt J., Weinberg R. S., Breckon J. D., Claytor R. P. (2011). Adolescent physical activity participation and motivational determinants across gender, age, and race. *Journal of Physical Activity & Health*.

[71] Mersh R., Fairclough S. J. (2010). Physical activity, lesson context and teacher behaviours within the revised English National Curriculum for Physical Education: A case study of one school. *European Physical Education Review*.

[72] Løndal K. (2011). Bodily Play in the after-School Program: Fulfillment of intentionality in interaction between body and place. *American Journal of Play*.

[73] Slingerland M., Haerens L., Cardon G., Borghouts L. (2014). Differences in perceived competence and physical activity levels during single-gender modified basketball game play in middle school physical education. *European Physical Education Review*.

[74] Tonge K. L., Jones R. A., Okely A. D. (2016). Correlates of children's objectively measured physical activity and sedentary behavior in early childhood education and care services: A systematic review. *Preventive Medicine*.

[75] Pasek M., Michalowska-Sawczyn M., Nowak-Zaleska A. (2014). Changes in maximal aerobic fitness and students attitude towards physical effort during outdoor and indoor school lessons of physical education. *Baltic Journal of Health Physical Activity*.

[76] Eccles J., Adler T. F., Futterman R. (1983). Expectancies, values, and academic behaviors. *Achievement and Achievement Motives*.

