# Supplementary material for: Correlates of Physical Activity of Students in Secondary School Physical Education: A Systematic Review of Literature
Source: Biomed Res Int. 2019 Feb 19;2019:4563484. doi: 10.1155/2019/4563484 (PMC6399562; doi:10.1155/2019/4563484)
Supplement: Supplementary Materials — Table S1: a record of the search strategy used for each database. Table S2: methodological quality assessment items. Table S3: methodological quality assessment per quality item and per study. [file 4563484.f1.docx]

**Table S1. Search strategies**

| **Database: ERIC**  **Number of results:** **12,093**  ((physical education OR PE OR lesson* OR class*) AND (youth* OR adolescen* OR young* OR teenager* OR primary OR elementary OR middle OR high OR secondary OR school OR studen*) AND (physical activity OR physically active OR sport OR exercise* OR activity level OR motor activi*)) in Title, Abstract, Keywords.  Filters: English |
| --- |
| **Database: SPORTDISCUS**  **Number of results: 10,495**  ((physical education OR PE OR lesson* OR class*) AND (youth OR adolescen* OR young* OR teenager* OR primary OR elementary OR middle OR high OR secondary OR school OR studen*) AND (physical activity OR physically active OR sport OR exercise* OR activity level OR motor activi*)) in Title, Abstract, Keywords.  Filters: English |
| **Database: PubMed**  **Number of results: 1,725**  Filters: English  ((physical education[Title/Abstract]) OR PE[Title/Abstract]) OR lesson[MeSH Terms]) AND (elementary[Title/Abstract]) OR primary[Title/Abstract]) OR middle[Title/Abstract]) OR high[Title/Abstract]) OR secondary[Title/Abstract]) OR school[Title/Abstract]) OR studen*[Title/Abstract]) OR adolescen*[MeSH Terms]) AND (physical activity[Title/Abstract]) OR physically active[Title/Abstract]) OR activity level[Title/Abstract]) OR exercise[MeSH Terms]))  Filters: English |
| **Database: PsycINFO**  **Number of results: 214**  ((physical education OR PE OR lesson* OR class*) AND (youth* OR adolescen* OR young* OR teenager* OR primary OR elementary OR middle OR high OR secondary OR school OR studen*) AND (physical activity OR physically active OR sport OR exercise* OR activity level OR motor activi*)) in Title, Abstract, Keywords.  Filters: English |
| **Database: Academic Search Premier**  **Number of results: 14,536**  ((physical education OR PE OR lesson* OR class*) AND (youth* OR adolescen* OR young* OR teenager* OR primary OR elementary OR middle OR high OR secondary OR school OR studen*) AND (physical activity OR physically active OR sport OR exercise* OR activity level OR motor activi*)) in Title, Abstract, Keywords.  Filters: English |
| **Database: Web of Science**  **Number of results: 535**  ((physical education OR PE OR lesson* OR class*) AND (youth* OR adolescen* OR young* OR teenager* OR primary OR elementary OR middle OR high OR secondary OR school OR studen*) AND (physical activity OR physically active OR sport OR exercise* OR activity level OR motor activi*)) in Title.  Filters: English |

Table S2. Methodological quality assessment items

| Item | Description |
| --- | --- |
| Design | 1. Did the study design a longitudinal/ prospective study? |
| Sample | 2. Did the study sample randomly selected? (or provided other data to indicate the representative of the study sample) |
|  | 3. Did the characteristics of the sample clearly described? |
| Study attrition | 4. Did the study report the numbers of individuals who completed each of the different measures and did participants complete at least 80% of measures for a cross-sectional study and 70% for a longitudinal study? |
| Data collection |  |
|  | 5. Did the study report a clear description of the outcome of physical activity, and did the instruments have acceptable quality? (validation in same age group published or validation data provided in the manuscript). |
|  | 6. Did related factors measurement use validated methods and describe details of assessment? (A clear description of related factors measurement was provided. The factors measurement should be used as a valid tool; sociodemographic factors should be present as appropriate cut-off points). |
|  |  |
| Data analysis | 7. Did the statistical analysis was appropriate and confounding variables accounted for in the analyses? |
|  | 8. Did the study report the power calculation and whether the power was adequately to detect the hypothesized relationship? |

Table S3. Methodological quality assessment per quality item and per study

| **No** | **Study design** | **Sample representative** | **Sample characteristics** | **Study attrition** | **Outcome measurement** | **Related factors measurement** | **Confounding variables** | **Power calculation** | **Total** |
| --- | --- | --- | --- | --- | --- | --- | --- | --- | --- |
| [2] | 0 | 0 | 1 | 0 | 1 | 1 | 1 | 0 | 4 |
| [10] | 0 | 1 | 1 | 0 | 1 | 1 | 1 | 0 | 5 |
| [11] | 0 | 1 | 1 | 0 | 1 | 1 | 1 | 1 | 6 |
| [23] | 0 | 1 | 0 | 1 | 1 | 1 | 1 | 0 | 5 |
| [24] | 0 | 1 | 1 | 1 | 1 | 1 | 1 | 0 | 6 |
| [25] | 0 | 1 | 1 | 0 | 1 | 1 | 1 | 0 | 5 |
| [26] | 0 | 1 | 1 | 0 | 1 | 0 | 1 | 0 | 4 |
| [27] | 0 | 1 | 0 | 1 | 1 | 0 | 1 | 0 | 4 |
| [28] | 0 | 0 | 0 | 1 | 1 | 1 | 1 | 0 | 4 |
| [29] | 0 | 0 | 1 | 0 | 1 | 1 | 1 | 0 | 4 |
| [30] | 0 | 1 | 0 | 0 | 1 | 0 | 1 | 1 | 4 |
| [31] | 0 | 1 | 0 | 1 | 0 | 1 | 1 | 0 | 4 |
| [32] | 0 | 0 | 1 | 1 | 1 | 1 | 1 | 0 | 5 |
| [33] | 0 | 0 | 1 | 0 | 1 | 1 | 1 | 1 | 5 |
| [34] | 0 | 0 | 1 | 0 | 1 | 0 | 1 | 1 | 4 |
| [35] | 0 | 1 | 1 | 0 | 1 | 1 | 1 | 1 | 6 |
| [36] | 1 | 0 | 1 | 0 | 1 | 1 | 1 | 0 | 5 |
| [37] | 0 | 0 | 1 | 0 | 1 | 0 | 1 | 1 | 4 |
| [38] | 0 | 0 | 1 | 0 | 1 | 0 | 1 | 1 | 4 |
| [39] | 0 | 0 | 1 | 1 | 1 | 1 | 1 | 0 | 5 |
| [40] | 0 | 0 | 1 | 0 | 1 | 1 | 1 | 1 | 5 |
| [41] | 0 | 0 | 1 | 0 | 1 | 1 | 1 | 0 | 4 |
| [42] | 0 | 0 | 1 | 1 | 1 | 1 | 1 | 0 | 5 |
| [43] | 0 | 0 | 1 | 0 | 1 | 1 | 1 | 0 | 4 |
| [44] | 0 | 0 | 1 | 0 | 1 | 1 | 1 | 0 | 4 |
| [45] | 0 | 1 | 0 | 0 | 1 | 1 | 1 | 0 | 4 |
| [46] | 0 | 1 | 1 | 0 | 1 | 1 | 1 | 0 | 5 |
| [47] | 0 | 1 | 1 | 1 | 1 | 1 | 1 | 0 | 6 |
| [48] | 0 | 1 | 1 | 0 | 1 | 1 | 1 | 1 | 6 |
| [49] | 0 | 0 | 1 | 0 | 1 | 1 | 1 | 0 | 4 |
| [50] | 0 | 1 | 1 | 0 | 1 | 1 | 1 | 1 | 6 |
| [51] | 0 | 0 | 1 | 0 | 1 | 1 | 1 | 0 | 4 |
| [52] | 0 | 0 | 1 | 0 | 1 | 1 | 1 | 0 | 4 |
| [53] | 0 | 0 | 1 | 1 | 1 | 0 | 1 | 1 | 5 |
| [54] | 1 | 0 | 1 | 1 | 1 | 1 | 1 | 1 | 7 |
| [55] | 0 | 0 | 1 | 0 | 1 | 1 | 1 | 1 | 5 |
| [56] | 0 | 0 | 1 | 0 | 1 | 1 | 1 | 1 | 5 |
| [57] | 0 | 1 | 1 | 0 | 1 | 1 | 0 | 0 | 4 |
| [58] | 1 | 0 | 1 | 1 | 1 | 1 | 1 | 0 | 6 |
| [59] | 1 | 0 | 1 | 1 | 1 | 1 | 1 | 0 | 6 |
| [60] | 0 | 0 | 1 | 0 | 1 | 1 | 1 | 0 | 4 |
| [61] | 1 | 0 | 1 | 0 | 1 | 1 | 0 | 0 | 4 |
| [62] | 0 | 0 | 1 | 0 | 1 | 1 | 1 | 0 | 4 |
